# Illinois State Medical Society

**Published:** 1858-09

**Authors:** 


ILLINOIS STATE MEDICAL SOCIETY.

Some members of the State Medical Society have recently made inquiries in reference to the *Transactions*. For the information of all such, we would state that so few of the members have paid the annual assessment, as to leave only about fifteen dollars in the treasury after paying the arrearages of last year. Of course, the Secretary cannot publish the *Transactions* with such a state of the treasury, unless he advances the printer's pay out of his own pocket. Will those members who are delinquent take the hint? Dr. J. W. Freer, of Chicago, is the Treasurer, and the amount of the annual assessment is *three* dollars.

